# Data mining process for predicting diabetes mellitus based model about other chronic diseases: a case study of the northwestern part of Nigeria

**DOI:** 10.1049/htl.2018.5111

**Published:** 2019-07-09

**Authors:** Muhammad Musa Uba, Ren Jiadong, Muhammad Noman Sohail, Muhammad Irshad, Kaifei Yu

**Affiliations:** 1 Department of Information Sciences and Technology, Yanshan University, Qinhuangdao, Hebei 066000, People's Republic of China; 2 Department of Electrical Engineering and Control, Yanshan University, Qinhuangdao, Hebei 066000, People's Republic of China

**Keywords:** regression analysis, pattern classification, diseases, medical disorders, medical computing, data mining, patient treatment, medical diagnostic computing, patient diagnosis, biomedical measurement, testing sets, training data, best-fitted model converges, predicted model, diabetic patients, chronic diseases, data mining process, diabetes mellitus based model, northwestern part, Nigeria, diabetes mellitus model data mining based approaches, dataset, seven northwestern states, primary sources, secondary sources, diabetic mellitus, hospital data, DM techniques, confusion matrix, correlation coefficient

## Abstract

To predict diabetes mellitus model data mining (DM) based approaches on the dataset collected from the seven northwestern states of Nigeria. Data were collected from both primary and secondary sources through questionnaires and verbal interviews from patients with diabetic mellitus and other chronic diseases. Some hospital data were also used from the records of patients involved in this work. The dataset comprises 281 instances with 8 attributes. R programming software (version 5.3.1) was used in the experiments. The DM techniques used in this research were binomial logistic regression, classification, confusion matrix and correlation coefficient. The data were partitioned into training and testing sets. Training data were used in building the model while testing data were used to validate the model. The algorithm for the best-fitted model converges with null deviance: 281.951, residual deviance: 16.476 and AIC: 30.476. The significance variables are AGE, GLU, DBP and KDYP with 0.025, 0.01, 0.05 and 0.025 *P* values, respectively. The predicted model accounted for the accuracy of ∼97.1%. The correlation analysis results revealed that diabetic patients are more likely to be hypertensive than patients with other chronic diseases considered in the research.

## 1 Introduction

The recent developments in biotechnology and health sciences have led to a significant production of data, such as clinical information, generated from large Electronic Health Records. This information can be used to forecast and scrutinise health care ratios of the entire population. By using Data Mining (DM) techniques, it is possible to extract hidden and useful information from datasets known as Knowledge Discovery in Database and Computer-based information system [1].

Logistic regression is a statistical technique used in predicting the probability of an event given a set of predictor variables. The procedure is more sophisticated than linear regression procedures. Binary logistic regression procedure empowers one to decide on the predictive model using binary dependent variables. It explains the relationship between a binary dependent variable and a set of independent variables. Independent variables can be continuous or discrete. Logistic regression as a non-linear regression model is a special case of the generalised linear model (GLM) [2] where the assumption of normality and constant variance of residuals is not satisfied. Logistic regression models have demonstrated their precision in many classification frameworks [3].

The significance of DM in health sector elevates further challenges, which entails explicit processes and tools. Cross-domain knowledge is of paramount importance to accomplish practical results. The brisk evolution in the automation of the healthcare industry gives a huge amount of heterogeneous, mutually structured and unstructured data accessible for research and secondary use. There are several algorithms implemented to categorise, bunch, and find hidden patterns in data. Domain-Specific issues of health care are yet to be resolved. As discussed by Abidi and Hoe [4], particular problems have been resolved in the effective appliance of DM systems. According to their studies, besides resolving depersonalisation, multi-relational and media data pre-processing clinical data heterogeneity, and quality issues, the DM process is sub-optimal or infeasible.

Diabetes is a persistent health problem and pandemics. In developing countries, customary tribal societies are adopting a contemporary lifestyle, while developing continual health problems usually associated with developing nations [5]. The direct and indirect problems caused by the disease surpassed the financial and human resources of the health care system in sub-Saharan Africa (SSA) [6]. Presently, hypertension, diabetes, and coronary artery diseases are among the foremost continual health conditions observed in SSA [7].

The projected predominance of diabetes in Africa is 1% in rural areas and 7% in urban SSA [8], while the incidence in Nigeria varies from 0.65% in rural areas to 11% in urban areas. Data from the World Health Organization (WHO) reported that Nigeria has the greatest number of people living with diabetes in Africa [9]. Nigeria, as the most densely populated countries in Africa, has approximately 196 million people in a million km^2^ area. Nigeria is also the seventh leading population in the world [10]. According to the United Nations, Nigeria's population will attain 411 million by 2050. Nigeria may then be the third most populous country in the world. In 2100, the population of Nigeria may reach 794 million [11]. The northwestern region is the second largest geopolitical area, covering 216,065 km^2^ and the most densely populated areas with an estimated population of 45 million people [12].

Recently, researches on diabetes in Nigeria were conducted with a plan to investigate and evaluate the incidence of diabetes among different social and economic groups in Port Harcourt. The model for Nigerians may emerge and be able to ascertain whether or not those with high blood glucose are aware of their diabetic problem [13]. In 2008, the benchmark for diabetes studies [14] was conducted athwart some selected Health centres in Nigeria, with objectives, clinical and laboratory profile evaluating the eminence of care of Nigerians diabetics with a view to planning and improving diabetes care. Another related study was carried out in northwestern Nigeria to assess diabetic patients’ compliance of the management, including Socio-demographic factors influencing their conformity [15]. However, in spite of the growing prevalence of diabetes mellitus and other chronic diseases, particularly in northwestern Nigeria, to the best of my knowledge, there has been a paucity of research and awareness in the area. Given this, a research was intended to be carried out to predict diabetes mellitus models about other chronic diseases using DM-based approaches, and northwestern part of Nigeria as the case study.

## 2 Material and methods

This section seeks to explain the analytical platform and the methods used in this Letter. This consists of sampling techniques used, dataset description, collection, issue of ethical approval and the software programming language used for the experiments. The DM techniques used include binomial logistic regression, classification and confusion matrix, and correlation coefficient.

### 2.1 Proposed analytical platforms for the model

**Fig. 1:**
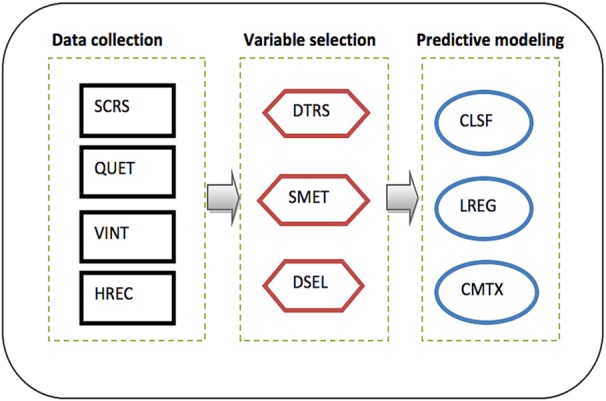
Proposed analytical platform of the predicted model Legend: SCRS: Systematic cluster random sampling, QUET: Questionnaire, VINT: Verbal interview, HREC: Hospital record, DTRS: Data transformation, SMET: Stepwise method, DSEL: Data selection, CLSF: Classification, LREG: Logistic regression and CMTX: Confusion matrix

The proposed analytical platform of the predicted model is presented in Fig. 1, in three stages. Stage 1: procedure and method used in data collection. Stage 2: processes of variable selections. Stage 3: the methods used in predicting the model.

### 2.2 Data description

#### 2.2.1 Ethical approval

All the study procedures performed were, according to the Helsinki Declaration and ethical approval obtained from the Yanshan University ethical board.

#### 2.2.2 Sampling techniques used in the process of data collection

The sampling techniques in distributing questionnaires and determining the sample size used in this paper are both combined probability and non-probability sampling techniques. A probability cluster sampling was applied at the beginning where the entire population was divided into groups or clusters; it's related to the selection of a subset of individuals from the population to estimate the characteristics of the entire population [27]. Each attribute determines one or more properties of the observable subjects distinguished as independent individuals. On the other hand, a non-probability sampling technique was used by the researcher to select samples based on subjective judgment rather than random selection. A convenience sampling process by which samples are drawn from the population because they are conveniently available to the researcher also employed.

The entire northwestern part of Nigeria comprises of seven (7) states. Each State was divided into three (3) clusters according to their senatorial zones (i.e. South, central and north). Government-owned hospitals were chosen in each cluster of the six (6) states, while in Kano, the number of the hospitals was doubled due to its population. Our target population is diabetes and other chronic disease patients. To achieve greater precision in the data collection, the author decided to distribute the entire questionnaire by himself across the states as well as interview the patients with the help of some hospital staff.

#### 2.2.3 Data collection

Data were collected from both primary and secondary sources through questionnaires and verbal interviews from patients who have diabetes mellitus and other chronic diseases. Some part of the hospital data were also used from the record departments of all hospitals under our study. The dataset comprises 281 instances with 8 attributes for this particular study. The attributes were abbreviated as; diabetes mellitus patient's (TYPE), patient's age (AGE), patient's glucose level (GLU), patient's diastolic blood pressure (DBP), a patient's body mass index (BMI), Symptoms related to kidney problems (KDYP), Symptoms related to heart/cardiovascular problems (HETP) and Symptoms related to eye problems (EYEP).

#### 2.2.4 Attribute information

TYPE – Patient's diabetes status (binary yes or no).
AGE – Patient's age (numeric: from 14 to 85).
GLU – Patient's glucose level (numeric with range: 3.9–7.2 mmol/l normal and >7.2 mmol/l diabetic).
DBP – Patient's diastolic blood pressure (numeric: <80 mmHg normal, 80–120 mmHg hypertensive and >120 mmHg crisis).
BMI – Patient's body mass index (numeric: <18.5 kg/m^2^ underweight, from 18.5 to 25 kg/m^2^ normal, 25 to 30 kg/m^2^ over weight and >30 kg/m^2^ obese).
KDYP – Symptoms related to kidney problems (binary yes or no).
HETP – Symptoms related to heart/cardiovascular problems (binary yes or no).
EYEP – Symptoms related to eye problems (binary yes or no).

**Fig. 2:**
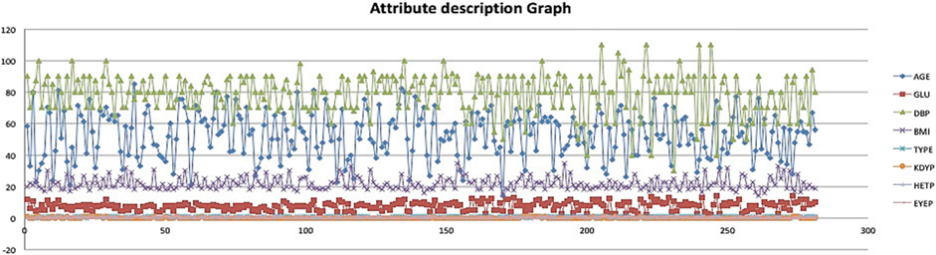
Attribute details in a graph format that has been used while the study conduct

The details of attributes can be seen in Fig. 2.

### 2.3 Variable selections

Variable selection is a process of selecting leading variables from the datasets and removing unrelated features, concerning the task to be performed. The purpose consists of identifying a set of *P* process variables, *P < J* able to better explain and envisage the response variable *y* [16]. Stepwise variable selection manner is a recipe of backward elimination and forward selection processes. It addresses both processes, based on the significance of score statistics, and the probability of likelihood-ratio statistics on the conditional parameter estimates. Variables can be removed, added or changed in the processes at each stage. Akaike information criterion (AIC) was used to check model adequacy.

$${\rm AIC = } - 2{\rm log - likelihood} + 2P\comma \; {\rm where}\, P\, {\rm is}\, {\rm the\, potential\, predictors}.$$

### 2.4 Binomial logistic regression model

Is a special case of a GLM dealing with modelling of binary responses. Let's consider a random variable *L* that can take on one of two possible values. Given a dataset with a total sample size of *M*, where each observation is independent, ***L*** can be considered as a column vector of *M* binomial random variables $L_i$. By convention, a value of 1 is used to indicate ‘success’ and a value of 0 used to signify ‘failure.’ To simplify computational details of estimation, it is convenient to aggregate the data such that each row represents one distinct combination of values of the independent variables. These rows are often referred to as ‘populations.’ Let *N* represent the total number of populations and let ***n*** be a column vector with an element $n_i$ representing the number of observations in a population $i = 1\comma \; \, \ldots \comma \; \, N$, where $\Sigma _{i = 1}^N n_i = M$ the total sample size. Now, let ***Y*** be a column vector of the length *N* where each element *Yi* is a random variable representing the number of successes of ***L*** for the population. Let the column vector ***y*** contain elements $y_i$ representing the observed counts of the number of successes for each population. Let ${\bi \theta }$ be a column vector also of the length *N* with elements $\theta _i = p\lpar l_i = 1/i\rpar $, i.e. the probability of success for any given observation in the *i*th population. The linear component of the model contains the design matrix and the vector of parameters to be estimated. The design matrix of independent variables, *X*, is composed of *N* rows and *K* + 1 columns, where *K* is the number of independent variables specified in the model, for each row of the design matrix, the first element $x_{io} = 1$. This is the intercept. The parameter vector, ***β***, is a column vector of the length *K* + 1. There is one parameter corresponding to each of the *K* columns of independent variable settings in *X*, plus one, $\beta _0$ for the intercept [17]. The transformed *logit*, which is the logistic regression model, equates the log-odds of the probability of success, to the linear component as:

(1) $$\log \left({\displaystyle{{\theta _i} \over {1 - \theta _i}}} \right)= \sum\limits_{k = 0}^K {x_{ik}\beta _k\quad i = 1\comma \; 2\comma \; ...\comma \; N} \eqno\lpar 1\rpar $$

where $\left({\theta _i/\lpar 1 - \theta _i\rpar } \right)$ is known as the odds of an event. Suppose *y* takes the values 1 for an event and 0 for a non-event, hence *y* has a Bernoulli distribution with probability parameter (and expected value) *p*.

### 2.5 Correlation coefficient

Is a statistical technique used to indicate the degree of the relationship between the variables. As well as the strength and direction of the relationship. The strength of the relationship can be a range from plus or minus ‘( + or − ) 1 to 0’, the stronger the relationship, the closer the value is to 1 [18]. The Pearson correlation coefficient equation is presented as follows:

(2) $$r = {\textstyle{{\sum\nolimits_{i = 1}^n {\lpar x_i - \overline x } \rpar \sum\nolimits_{i = 1}^n {\lpar y_i - \overline y } \rpar } \over {\sqrt {\sum\nolimits_{i = 1}^n {\lpar x_i - \overline x } \rpar ^2} \sum\nolimits_{i = 1}^n {\lpar y_i - \overline y } \rpar ^2}}}\eqno\lpar 2\rpar $$

### 2.6 Classification accuracy

As fundamental techniques for DM process [19], classification techniques can be used to create an idea of the type of customers, objects and items by specifying multiple attributes to specify the defined class. The main goal of classification is to assign a class to find previously unseen records as accurately as possible. If there is a collection of records (called a training set) and each record contains a set of attributes, then one of the attributes is a class [20, 21]. The motive is to find a classification model for class attributes, where a testing set is used to determine the accuracy of the model. The known figures set are divided into training and testing sets. Training sets are used to build the model and testing sets are used to validate it [22, 23]. Classification process consists of a training set that is analysed by classification algorithms and the classifier or learner [24]. Model is, therefore represented in the structure of classification rules [25]. Testing data is used in the classification rules to estimate accuracy. The initial model is represented in the form of classification rules, decision trees or mathematical formulas.

### 2.7 Confusion matrix

In predictive analytics, a table of confusion allows more detailed analysis than the percentage of classification (accuracy) in the field of machine learning and distinctively the problem of classification in statistics. A confusion matrix, also known as error matrix [4] is a specific table layout that permits visualisation of the feat of an algorithm, classically supervised learning. A confusion matrix is appraised of correct classifications, a 2 × 2 square matrix consists of true positive (TP), true negative (TN), false positive (FP) and false negative (FN). The accuracy is computed by taking the sum of the right diagonal element divided by the total sum of the entire observations as follows:

(3) $${\rm Accuracy} = \lpar {\rm TP} + {\rm TN}\rpar /\lpar {\rm TP} + {\rm TN} + {\rm FP} + {\rm FN}\rpar \eqno\lpar 3\rpar $$

### 2.8 R programming software

The programming software used for the experiments. Free open programming software used for programming, statistics and graphics, was developed by Ross Ihaka and Robert Gentleman at the University of Auckland in New Zealand. The name R came from their respective first names [26], can be accessed online via http://www.r-project.org.

## 3 Results and discussion

### 3.1 Results

Tables 1 and 2 represent the logistic regression model result of the R programming software algorithms, for diabetes patients (TYPE) as a response variable, while the remaining variables as explanatory variables.

**Table 1:** Logistic regression model result

Variables	Coefficients	Std error	*Z* values	*P* values
constant	−48.12040	21.61366	−2.226	0.0260*
AGE	0.30854	0.19877	1.552	0.0246*
GLU	6.52985	2.70314	2415	0.0157*
BMI	−0.13504	0.09778	−1.381	0.1673
DBP	−0.06805	0.17965	−0.379	0.7048
KDYP	7.97545	4.15712	1.919	0.0550^.^
HRTP	3.28995	3.06506	1.073	0.2831
EYEP	−1.68871	2.02458	−0.834	0.4042

Legends: AGE: Patients Age, GLU: Patents Glucose level, BMI: Patients Body Mass Index, DBP: Patients Diastolic Blood Pressure, KDYP: Symptoms related to kidney problems, HETP: Symptoms related to heart/cardiovascular problems and EYEP: Symptoms related to eye problems.

Null deviance: 281.951.

RD: 16.302.

AIC: 32.302.

Fisher's iterations: 12.

**Table 2:** Logistic regression model result

Variables	Coefficients	Std error	*Z* values	*P* values
constant	−38.26928	13.32100	−2.872	0.00407**
AGE	0.18394	0.08125	2.264	0.02358*
GLU	5.43762	1.89178	2.874	0.00405**
BMI	−0.12266	0.14977	−0.819	0.41280
DBP	−0.07408	0.05872	−1.262	0.05708^.^
KDYP	10.04632	4.23233	2.374	0.01761*

Legends: AGE: Patients Age, GLU: Patents Glucose level, BMI: Patients Body Mass Index, DBP: Patients Diastolic Blood Pressure and KDYP: Symptoms related to kidney problems.

Null deviance: 281.951.

RD: 16.476.

AIC: 30.476.

Fisher's iterations: 11.

Table 3 presents a confusion matrix for the actual and predicted values of the logistic regression results.

**Table 3:** Confusion matrix

Actual	Predicted	
	False	True
0	17	2
1	0	51

Accuracy = Sum of the right diagonal divided by the total sum of the entire observations = 0.971 = 97.1%.

Table 4 presents a correlation matrix describing the level of relationship between the variables TYPE, KDYP, HETP, EYEP and HBPK.

**Table 4:** Correlation matrix

	TYPE	KDYP	HETP	EYEP	HBPK
TYPE	1.00	0.09	0.08	0.13	0.25
KDYP	0.09	1.00	0.32	−0.01	0.09
HETP	0.08	0.32	1.00	0.11	0.02
EYEP	0.13	−0.01	0.11	1.00	0.09
HBPK	0.25	0.09	0.02	0.09	1.00

Legends: TYPE: Diabetes Patient's, KDYP: Symptoms related to kidney problems, HETP: Symptoms related to heart/cardiovascular problems and EYEP: Symptoms related to eye problems.

**Fig. 3:**
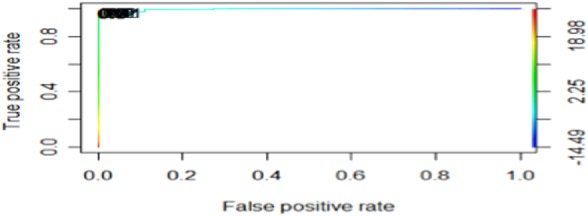
‘ROCR’ curve, for the TP and FP rates values

The ROC curve for the TP and FN values is shown in Fig. 3.

**Fig. 4:**
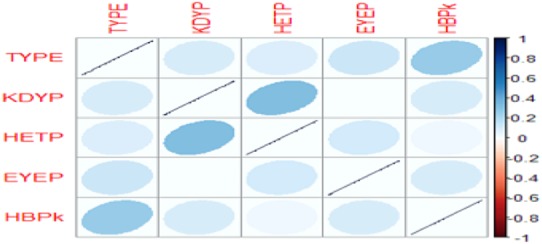
Correlation plot shows the graphical representation of correlations between the variables ‘TYPE’, ‘KDYP’, ‘HETP’, ‘EYEP’ and ‘HBPk’

The graphical representation of correlations between the variables TYPE, KDYP, HETP, EYEP and HBPK is shown in Fig. 4, from the correlation matrix given in Table 4. The darker ellipse indicates a strong positive correlation, while white shows no correlation.

### 3.2 Discussions

#### 3.2.1 Logistic regression

Tables 1 and 2 present logistic regression results for R algorithms, from the healthcare dataset with Eight (8) attributes; TYPE as the dependent variable and AGE, GLU, DBP, BMI, KDYP, HETP and EYEP as the independent variables. The data were partitioned into training and testing sets; the training part was used in building the model, while the testing part was used in validating the model.

In Table 1, the algorithms for the selection of factors with significance effect converged after 12 Fisher's iterations, variable AGE, GLU and KDYP were significant at 0.025, 0.025 and 0.05 *P* values, respectively, with the null deviance: 281.951, residual deviance (RD): 16.302 and AIC: 32.302.

In Table 2, less significant variables were removed by the model, after 11 Fisher's iterations the algorithms for selection of factors with significant effects converge with null deviance: 281.951, RD: 16.476 and AIC: 30.476, variables AGE, GLU, DBP and KDYP were significant at 0.025, 0.01, 0.05 and 0.025 *P* values, respectively, with the exception of BMI. Hence variable BMI will not be removed from the model.

#### 3.2.2 Confusion matrix

Table 3 presents a confusion matrix for the adequacy of the predicted model. The model predicted as follows: Seventeen (17) times the patient was actually diabetic; the model also predicts as diabetic. Fifty-one (51) times the patient was non-diabetic; the model also predicts as non-diabetic. Two (2) times the model was actually non-diabetic; the model predicts as diabetic (Type I) error. Zero (0) time the patient was actually diabetic; the model predicts as non-diabetic (Type II) error. This accounted for the model accuracy of ∼97.1%.

Fig. 3 presents the ROCR graph plot, for the confusion matrix TP rate and FP rate, for the prediction and performance of the model. Also was used to choose the threshold for the confusion matrix prediction, in the case that the default needs to be changed.

#### 3.2.3 Correlation analysis

Table 4 presents a correlation matrix for diabetes mellitus and some other chronic diseases. The correlation coefficient was used to check the relationship between them. It could be observed that the symptoms related to kidney problems have a relatively higher correlation with symptoms related to heart/cardiovascular problems (0.32), followed by diabetes mellitus and high blood pressure (0.25). There is a negative correlation between symptoms related to kidney problems and symptoms related to eye problems (−0.01). The observed correlation between diabetes mellitus and symptoms related to kidney problems is (0.09), diabetes mellitus and symptoms related to heart/cardiovascular problems are (0.08) and diabetes mellitus and symptoms related to eye problems (0.13). These are all positively weak.

Fig. 4 presents a pictorial representation of the correlation matrix. The darker the colour, the more strongly the relationship and vice-versa.

## 4 Conclusion

The result of this study builds a valid, adequate and comprehensive model for predicting diabetes mellitus about other chronic diseases. From the fitted models, the best model which describes the relationship between the variables with the highest precision in its algorithm converges to a minimal RD and AIC values of (16.764) and (30.764), respectively. Moreover, the model explains the accuracy level of 97.1% in the confusion matrix result.

Furthermore, from the correlation coefficient results, it has been revealed that diabetes mellitus patient is more likely to be hypertensive than the remaining chronic diseases.

## 9 Informed consents

Informed consent was obtained from all individual participants included in the study.
